# Burden of Anxiety Among School-Going Adolescents in Urban Bhopal: A Cross-Sectional Study

**DOI:** 10.7759/cureus.83186

**Published:** 2025-04-29

**Authors:** Darshan Parida, Archit Khardenavis, Swaha Pattanayak, Subba Krishna Nagaraj, Ashlesh Rupani, Sushma Yadav, Kritika Singhal, Ankur Joshi, Pankaj Prasad

**Affiliations:** 1 Community Medicine, Index Medical College, Hospital and Research Centre, Indore, IND; 2 Community and Family Medicine, All India Institute of Medical Sciences, Bhopal, IND; 3 Orthopedics, Fortis Memorial Research Institute, Gurugram, IND; 4 Community Medicine, Kanti Devi Medical College Hospital and Research Center, Mathura, IND; 5 Community and Family Medicine, All India Institute of Medical Sciences, Jodhpur, IND

**Keywords:** adolescents, anxiety, dass-42, mental health, psychological changes

## Abstract

Introduction: Adolescence is a critical developmental stage marked by physical, emotional, and psychological changes, making individuals vulnerable to mental health challenges, particularly anxiety. Anxiety during adolescence can significantly impact daily functioning, school performance, and relationships. In India, the prevalence of anxiety among adolescents is rising, exacerbated by academic pressures, social expectations, and limited mental health services. This study aimed to assess the prevalence of anxiety among school-going adolescents in Bhopal and to identify the associated factors.

Materials and methods: A community-based cross-sectional study was conducted from May 2019 to May 2021 among 1,500 adolescents aged 14-19 years who attended secondary and higher secondary schools in urban Bhopal, Madhya Pradesh, India. Multistage cluster sampling was used, and data were collected using the Depression, Anxiety, and Stress Scale (DASS-42), with a focus on the anxiety subscale. Statistical analyses, including descriptive statistics, chi-square tests, and logistic regression, were conducted to determine associations between anxiety levels and various demographic and socioeconomic factors using R software version 4.1.0 (R Foundation for Statistical Computing, Vienna, Austria, https://www.R-project.org/).

Results: The study found that 749 (53%) of adolescents experienced some level of anxiety. Among them, 150 (10.6%) had mild anxiety, 304 (21.5%) had moderate anxiety, 195 (13.8%) had severe anxiety, and 100 (7.1%) had extremely severe anxiety. No significant associations were found between anxiety and factors such as age, gender, or screen time. However, a statistically significant association was observed with the father’s occupation, where adolescents whose fathers were engaged in government or private services exhibited higher levels of anxiety (p = 0.007). Regression analysis revealed that gender, frequency of outdoor activities, and father's education level were not significantly associated with anxiety. However, paternal occupation showed significant effects, with participants whose fathers were in jobs other than business having lower odds of anxiety.

Conclusions: Anxiety is highly prevalent among school-going adolescents in Bhopal, with the father’s occupation being a significant determinant. Early identification and interventions involving both parents and teachers are crucial to addressing anxiety in adolescents. Schools should promote emotional well-being by establishing support systems and fostering open discussions about mental health.

## Introduction

Adolescence is a transformative phase where physical, emotional, and psychological changes converge, making adolescents susceptible to a range of psychosocial challenges. Among these, anxiety is one of the most common and concerning mental health issues. It is characterized by feelings of worry, nervousness, or unease, which can significantly affect an adolescent's daily functioning, school performance, and interpersonal relationships. Anxiety disorders are typically defined as excessive fear or worry that is difficult to control, lasting for at least six months, and often accompanied by symptoms such as restlessness, fatigue, difficulty concentrating, irritability, muscle tension, and sleep disturbances. Generalized anxiety disorder (GAD), which is one of the most common subtypes, involves persistent and excessive worry about various domains of life and is diagnosed based on Diagnostic and Statistical Manual of Mental Disorders, Fifth Edition (DSM-5) or International Classification of Diseases, 10th Revision (ICD-10) criteria. Anxiety during adolescence may be triggered by multiple factors, including academic pressure, peer relationships, family expectations, and the ongoing physiological changes that mark this developmental stage. Along with other mental health conditions like depression and stress, anxiety can substantially hinder an adolescent’s well-being, leading to long-term consequences if not addressed [1-3].

Globally, anxiety disorders are one of the leading causes of illness and disability among adolescents. Studies suggest that 10% to 20% of adolescents worldwide experience some form of mental health issue, including anxiety, with a notable prevalence among those aged 15-19 years [4,5]. In countries like the USA and Australia, approximately one in five adolescents suffers from mental health problems. In contrast, the prevalence in developing countries, including India, ranges between 12% and 29%, reflecting a rising concern in regions with limited mental health services [6].

India, with its vast adolescent population of approximately 243 million, faces a significant burden of anxiety and other mental health issues, as recent studies indicate that one out of every six Indian adolescents experiences mental illnesses, including anxiety disorders. This figure is alarming given the lack of adequate mental health infrastructure [6]. Academic pressures, heightened competition, social comparison, and family dynamics are among the many factors contributing to the increasing incidence of anxiety among school-going adolescents in India. In an educational system where academic excellence is often prioritized over emotional well-being, adolescents are more prone to experiencing anxiety, which often goes unrecognized and untreated [1].

Anxiety disorders, if left unmanaged, can extend into adulthood, leading to chronic mental health conditions that impair physical, emotional, and psychosocial functioning. Adolescents with unmanaged anxiety may face challenges in forming relationships, achieving academic goals, and engaging in social activities, which can limit their opportunities in later life [4]. Early detection and intervention are crucial, as they can significantly reduce the long-term burden of psychiatric disorders. Unfortunately, the current treatment modalities for anxiety and other related disorders remain limited in their scope and effectiveness. Hence, prevention strategies are often the most effective way to address this issue [5].

There's ample evidence that well-designed prevention programs can lower anxiety risks while strengthening resilience and healthy coping strategies in adolescents [6]. In India, government efforts, such as the Rashtriya Kishor Swasthya Karyakram, aim to enhance support through adolescent-friendly health clinics and programs focused on adolescent reproductive and sexual health. These programs provide safe spaces for young people to discuss their mental health, including anxiety [7]. Still, many of these services tend to cluster in urban centers, leaving rural adolescents with limited access. Moreover, mental health issues often receive less attention compared to reproductive health and nutrition, and counseling opportunities remain scarce for many [8].

Given these challenges, it is imperative to develop comprehensive strategies that focus on early detection, prevention, and management of anxiety among adolescents. Schools can play a pivotal role in this effort by creating awareness, reducing stigma, and providing support systems that help adolescents cope with anxiety. Mental health programs tailored to the needs of adolescents, including school-based interventions and community outreach initiatives, are essential for reducing the overall burden of anxiety in this population.

This study, therefore, seeks to contribute to the growing body of research by quantifying the current burden of anxiety among school-going adolescents in Bhopal and identifying potential correlates of anxiety, such as family dynamics, peer relationships, and academic pressures. The study aimed to determine the prevalence of anxiety among school-going adolescents and to identify factors associated with anxiety through the use of the Depression Anxiety Stress Scale (DASS-42) questionnaire.

## Materials and methods

Study design

This study employed a community-based, cross-sectional design to determine the prevalence and correlates of anxiety among school-going adolescents in urban areas of Bhopal.

Study setting and duration

The study was conducted in the urban areas of Bhopal, Madhya Pradesh. Schools in the areas covered by the Centre for Urban Health of the All India Institute of Medical Sciences, Bhopal, a tertiary healthcare center, were selected for the study. These regions offered operational feasibility and integration with existing health services. The selection of schools in these areas provided a representative sample of adolescents from different socioeconomic backgrounds. The study was conducted for two years, from 04 May 2019 to 01 May 2021.

Sample size

The sample size was calculated using the formula for simple random sampling (SRS):

\[ \frac{Z^2 \times P(1 - P)}{D^2} \]

where Z = 1.96 for 95% confidence level, P = 28% (based on previous literature), and D = 5% of P (i.e., 1.4). This yielded a sample size of approximately 310 for SRS. Since a multistage cluster sampling method was used, the sample size was adjusted based on the design effect. A pilot study was conducted among 15 sections, and based on the observed cluster variability, the design effect was calculated to be 4.37. Therefore, the adjusted sample size was

\[ N = 310 \times 4.37 = 1356 \]

To account for non-response and dropouts, the final sample size was rounded to 1500 adolescents.

Sampling method

The study used a multistage cluster sampling method. The target population included adolescents aged 14 to 19 who attended secondary and higher secondary schools (classes 9th to 12th). A section of a class was considered a cluster for sampling, ensuring that the selected students were representative of the larger adolescent population in Bhopal. A list of secondary and senior secondary schools within the coverage area of the Centre for Urban Health at the All India Institute of Medical Sciences, Bhopal, was created. From each school, 30 students per class (one section from classes 9th to 12th each) were selected using stratified random sampling. Thus, a total of 52 sections (1500/30=50 rounded off to 52 clusters) across all grades and 13 schools were included, ensuring a diverse representation of the adolescent population. Consent was obtained from the school principals, parents, and students. The participants were provided with a participant information sheet and a consent form, which needed to be signed by their parents and returned the following day. Students who met the inclusion criteria (aged 14 to 19 years and attending classes 9th to 12th) and who returned signed consent forms were included.

Participants self-reported any prior psychiatric diagnoses (including anxiety) on the study intake form, which was then confirmed via parent/guardian medical history and school health records; anyone with a documented or self-reported psychiatric illness was excluded. Those who did not consent were also excluded from the study. Thus, two students were excluded based on pre-existing psychiatric conditions and 33 based on non-consent.

Data collection

The administered study tool, available in both English and Hindi, consisted of two sections. The first section included questions based on sociodemographic details, physical activity, history of failure in a previous class, screen time (categorized as low when the total time spent using electronic devices such as TVs, laptops, mobile phones, tablets, etc., was ≤2 hours per day and high when it was >2 hours per day), and other related factors. The second section included the DASS-42, which was used as the primary tool for measuring anxiety. The DASS-42 is a validated questionnaire available in both English and Hindi, containing a specific subset of questions focused on anxiety. The DASS-42 anxiety subscale comprises 14 items, each scored from 0 (“did not apply to me at all”) to 3 (“applied to me very much or most of the time”), for a total score range of 0-42; scores are interpreted as normal (0-7), mild (8-9), moderate (10-14), severe (15-19), and extremely severe (≥20), and assess symptoms such as nervousness, worry, and physiological arousal [9].

The questionnaire was administered in the classroom setting, with students providing self-reported answers. Special care was taken to ensure privacy and avoid any peer pressure that might influence the responses. Students were encouraged to be honest in their answers, and steps were taken to maintain confidentiality.

Statistical analysis

After data collection, the responses were entered into Microsoft Excel (Microsoft Corporation, Redmond, WA, USA) and analyzed using R software version 4.1.0 (R Foundation for Statistical Computing, Vienna, Austria, https://www.R-project.org/). Descriptive statistics were used to summarize the anxiety scores, with measures such as mean, median, and standard deviation providing insights into the central tendencies and variability of anxiety levels among the participants. Cross-tabulations were performed to examine the association between anxiety levels and demographic factors such as age, gender, socioeconomic status, screen time, academic performance, and physical activity. Chi-square tests were used to assess the relationships between anxiety (as a categorical variable) and other categorical variables like gender or academic performance. A p-value of <0.05 was considered statistically significant for all tests. To identify independent predictors of anxiety, a logistic regression model was employed. The dependent variable was the presence or absence of anxiety, as determined by DASS-42 scoring, while the independent variables included demographic factors, socioeconomic status, academic performance, and other psychosocial correlates.

Ethical consideration

The study was conducted after obtaining ethical clearance from the Institutional Human Ethics Committee of All India Institute of Medical Sciences, Bhopal (approval number: IHEC-LOP/2019/MD0058, approval date: 3rd May 2019).

## Results

**Figure 1:**
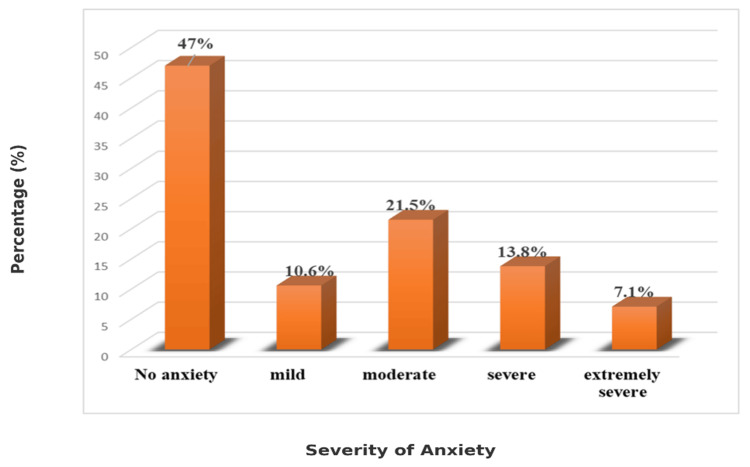
Prevalence of anxiety among study participants

The study included 1,500 participants; however, 88 were excluded due to incomplete data, resulting in a final analysis of 1,412 participants. For context, anxiety severity was classified using the DASS-42 anxiety subscale cut-offs: normal (0-7), mild (8-9), moderate (10-14), severe (15-19), and extremely severe (≥20). The mean age of the study participants was 16.46 ± 1.22 years. A total of 814 males (57.6%) and 598 females (42.4%) were included. Figure 1 shows that 47% (663) of the participants do not have any anxiety, 10.6% (150) were screened to have mild anxiety, 21.5% (304) were screened to have moderate anxiety, 13.8% (195) had severe anxiety, and the rest, 7.1%, of participants had extremely severe anxiety.

**Figure 2:**
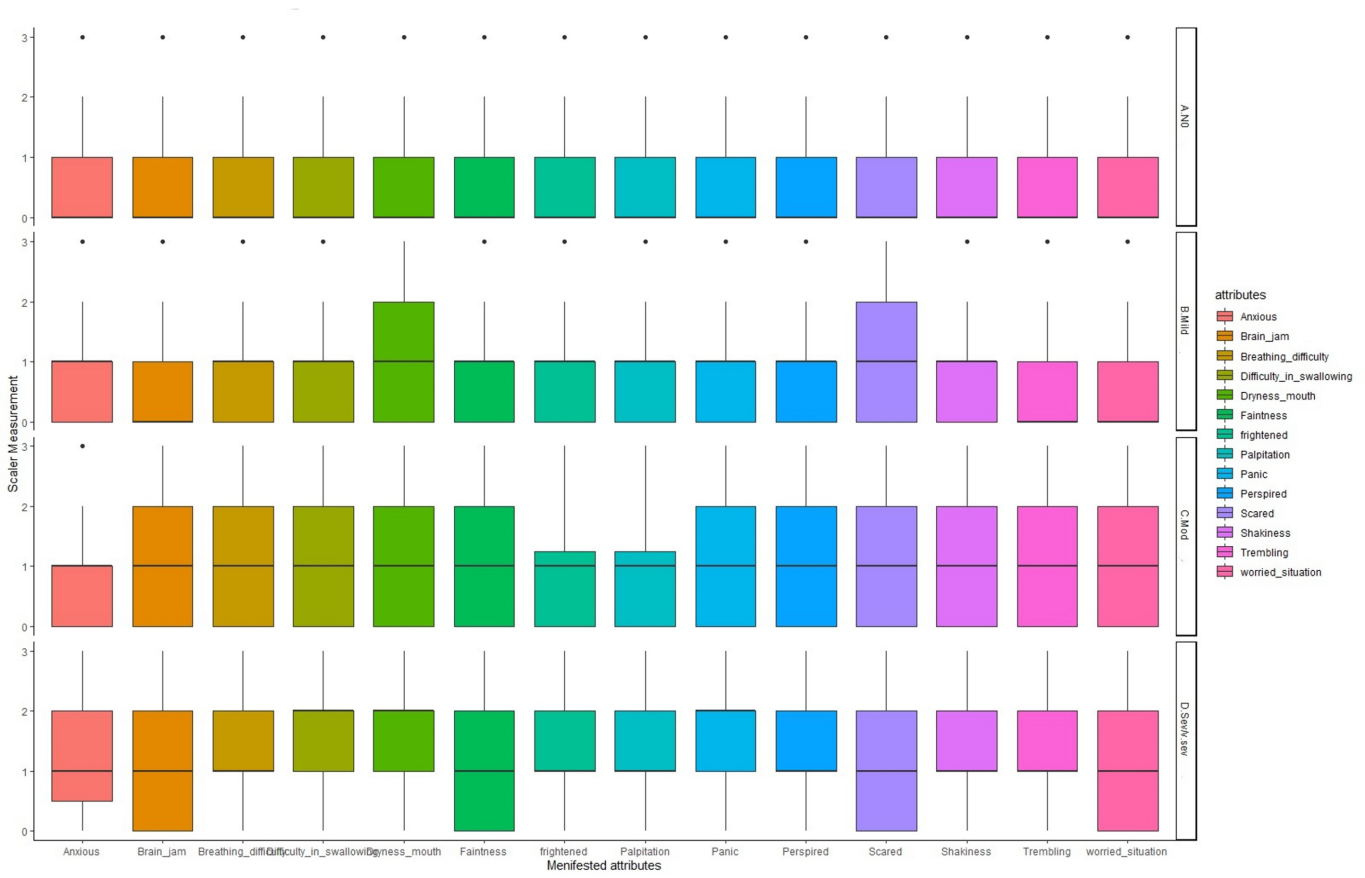
Anxiety attributes as per anxiety category

Figure 2 presents the box plot distribution of attributes of anxiety according to the severity level of anxiety. It was observed that participants with mild anxiety were more scared and had dry mouth as compared to any other symptoms. Similarly, with moderate anxiety, participants showed all other symptoms but less of being anxious, frightened, or having palpitations. Meanwhile, participants with severe anxiety complained of brain-jam, faintness, being scared, and being in a worried state, more so than other manifested symptoms of anxiety.

Table 1 shows the factors influencing the occurrence of anxiety among the participants. It revealed no significant association between anxiety and age groups (p = 0.923), gender (p = 0.532), class of study (p = 0.959), outdoor activity (p = 0.995), or educational background of parents (p = 0.163 for father’s education and p = 0.310 for mother’s education). The highest prevalence of anxiety was observed in the 16-17 age group (48%) and among males (57%), though these findings were not statistically significant. However, a significant association was found between anxiety and the father’s occupation (p = 0.020). Adolescents whose fathers were engaged in government or private services had higher levels of anxiety compared to those whose fathers were in business or other occupations. There was no significant difference in anxiety levels based on mother’s occupation (p = 0.073), screen time (p = 0.709), family type (p = 0.139), or socioeconomic status (p = 0.297). The results indicated that, while some factors like father’s occupation had a notable impact, most demographic and socioeconomic variables did not show a statistically significant influence on anxiety levels.

**Table 1:** Factors influencing the occurrence of anxiety Data presented as frequency (%); p-values derived from chi-square tests comparing characteristics between groups with and without anxiety; p<0.05 is considered significant

Characteristic	Anxiety present, N = 749	Anxiety not present, N = 663	Chi-square value	p-value
Age group				
14-15 yrs	192 (25.6%)	175 (26.4%)		
			16-17 yrs		360 (48%)	312 (47%)	0.16	0.923
>17 yrs	197 (26.3%)	176 (26.6%)						
Gender			0.4	0.532
Female	323 (43%)	275 (41%)		
Male	426 (57%)	388 (59%)		
Class of study				0.959
9^th^ class	192 (26%)	176 (27%)		
10^th^ class	173 (23%)	153 (23%)	0.3	
11^th^ class	187 (25%)	158 (24%)		
12^th^ class	197 (26%)	176 (27%)		
Outdoor activity				0.995
<1 day/week	115 (15%)	102 (15%)		
1-4 days/week	301 (40%)	268 (40%)	0.01	
≥5 days a week	333 (44%)	293 (44%)		
Ever failed resulting in a year back				0.475
Yes	11 (1.5%)	13 (2.0%)	0.51	
No	738 (99%)	650 (98%)		
Father education				0.163
Upto middle	7 (0.9%)	13 (2.0%)		
Upto intermediate	95 (13%)	72 (11%)	3.63	
Graduate	647 (86%)	578 (87%)		
Mother education				0.310
Upto middle	81 (11%)	85 (13%)	2.34	
Upto intermediate	201 (27%)	189 (29%)		
Graduate	467 (62%)	389 (59%)		
Father occupation				0.020
Professional	239 (32%)	205 (31%)		
Private/government service	318 (42%)	259 (39%)	9.82	
Business	130 (17%)	157 (24%)		
Others	62 (8.3%)	42 (6.3%)		
Mother occupation				0.073
Professional	92 (12.3%)	93 (14%)		
Private/government service	121 (16.1%)	80 (12%)	8.57	
Business	13 (1.7%)	7 (1.1%)		
Others	64 (8.5%)	74 (11.1%)		
Homemaker	459 (61.3%)	409 (61.7%)		
Screen time				0.709
Low (<2 hrs)	413 (55.1%)	359 (54.1%)	0.14	
High (>2 hrs)	336 (44.9%)	304 (45.9%)		
Family type				0.139
Nuclear	496 (66.2%)	414 (62.4%)	2.19	
Joint	253 (33.8%)	249 (37.6%)		
Socioeconomic status				0.297
Upper I	515 (68.7%)	431 (65%)		
Upper middle II	155 (20.7%)	166 (25%)	4.91	
Lower middle III	57 (7.6%)	43 (6.5%)		
Upper lower IV	21 (2.8%)	21 (3.1%)		
Lower V	1 (0.1%)	2 (0.3%)		

Table 2 shows the factors influencing various levels of anxiety among the participants, which revealed no statistically significant associations between most demographic and socioeconomic factors. Anxiety was found to be more prevalent in the 16-17 age group, with 47% of participants experiencing moderate anxiety and 49.8% experiencing severe anxiety, though this finding was not significant (p = 0.749). Similarly, gender did not significantly influence anxiety levels, with 56% of males and 44% of females experiencing moderate to severe anxiety (p = 0.836). Other factors such as class of study, outdoor activity, parents' education, and occupation also did not show significant associations with anxiety levels. The majority of participants, regardless of socioeconomic status or family type, experienced moderate to severe levels of anxiety. For instance, among participants from nuclear families, 68.8% experienced severe anxiety, but this was not statistically significant (p = 0.302). Furthermore, screen time (p = 0.967) and outdoor activity (p = 0.983) did not have a significant impact on anxiety severity.

**Table 2:** Factors influencing the various levels of anxiety Data presented as frequency (%); p-values derived from chi-square tests comparing characteristics across different anxiety severity groups; p<0.05 is considered significant

Characteristic	No anxiety, N = 663	Mild anxiety, N = 150	Moderate anxiety, N = 304	Severe anxiety, N = 295	Chi-square value	p-value
Age group						
14-15 yrs	175 (26.4%)	35 (23.3%)	87 (28.6%)	70 (23.7%)	3.46	
16-17 yrs	312 (47%)	70 (46.6%)	143 (47%)	147 (49.8%)		0.749
>17 yrs	176 (26.6%)	45 (30%)	74 (24.3%)	78 (26.4%)		
Gender						0.836
Female	275 (41%)	61 (41%)	133 (44%)	129 (44%)	0.85	
Male	388 (59%)	89 (59%)	171 (56%)	166 (56%)		
Class of study						0.917
9^th^ class	176 (27%)	35 (23%)	87 (29%)	70 (24%)		
10^th^ class	153 (23%)	32 (21%)	68 (22%)	73 (25%)	3.92	
11^th^ class	158 (24%)	38 (25%)	75 (25%)	74 (25%)		
12^th^ class	176 (27%)	45 (30%)	74 (24%)	78 (26%)		
Outdoor activity						0.983
<1 day/week	102 (15%)	26 (17%)	43 (14%)	46 (16%)		
1-4 days/week	268 (40%)	59 (39%)	121 (40%)	121 (41%)	1.07	
≥5 days a week	293 (44%)	65 (43%)	140 (46%)	128 (43%)		
Ever failed resulting in a year back						0.408
Yes	13 (2.0%)	2 (1.3%)	7 (2.3%)	2 (0.7%)	2.89	
No	650 (98%)	148 (99%)	297 (98%)	293 (99%)		
Father education						0.549
Upto middle	13 (2.0%)	0 (0%)	3 (1.0%)	4 (1.4%)	4.96	
Upto intermediate	72 (11%)	19 (13%)	39 (13%)	37 (13%)		
Graduate	578 (87%)	131 (87%)	262 (86%)	254 (86%)		
Mother education						0.353
Upto middle	85 (13%)	21 (14%)	31 (10%)	29 (9.8%)	6.66	
Upto intermediate	189 (29%)	32 (21%)	82 (27%)	87 (29%)		
Graduate	389 (59%)	97 (65%)	191 (63%)	179 (61%)		
Father occupation						0.146
Professional	205 (31%)	52 (35%)	98 (32%)	89 (30%)	13.38	
Private/government service	259 (39%)	60 (40%)	132 (43%)	126 (43%)		
Business	157 (24%)	22 (15%)	51 (17%)	57 (19%)		
Others	42 (6.3%)	16 (11%)	23 (7.6%)	23 (7.8%)		
Mother occupation						0.116
Professional	93 (14%)	10 (6.6%)	42 (13.8%)	40 (13.5%)		
Service	80 (12%)	26 (17.3%)	49 (16.1%)	46 (15.6%)	17.99	
Business	7 (1%)	4 (2.6%)	4 (1.3%)	5 (1.7%)		
Others	74 (11.1%)	8 (5.3%)	29 (9.5%)	27 (9.1%)		
Homemaker	409 (61.7%)	102 (68%)	180 (59.2%)	177 (60%)		
Socioeconomic status						0.671
Upper I	431 (65%)	99 (66%)	218 (71.7%)	198 (67.1%)		
Upper middle II	166 (25%)	35 (23.3%)	58 (19.1%)	62 (21%)	9.37	
Lower middle III	43 (6.5%)	10 (6.6%)	21 (6.9%)	26 (8.8%)		
Upper lower IV	21 (3.1%)	6 (4%)	7 (2.3%)	8 (2.7%)		
Lower V	2 (0.3%)	0	0	1 (0.3%)		
Screen time						0.967
Low (<2 hrs)	359 (54.1%)	82 (54.6%)	166 (54.6%)	165 (55.9%)	0.26	
High (>2 hrs)	304 (45.9%)	68 (45.4%)	138 (45.4%)	130 (44.1%)		
Family type						0.302
Nuclear	414 (62.4%)	96 (64%)	197 (64.8%)	203 (68.8%)	3.64	
Joint	249 (37.6%)	54 (36%)	107 (35.2%)	92 (31.2%)		

Table 3 shows the interrelationship between various determinants that affect anxiety. It was observed that, by keeping female gender as the reference category, the odds of males suffering from anxiety are 1.07 times that of females; however, the p-value was found to be 0.532 (>0.05), which means that the finding was statistically insignificant. Similarly, by keeping the time spent outdoors as a reference category, it was observed that the odds of those engaged in outdoor activities for one to four days a week suffering from anxiety were 1.00 times higher than those with less than one day a week of outdoor activities; however, the p-value was found to be 0.981 (>0.05), which means that the finding was statistically insignificant. Likewise, the odds of participants who spent more than five days a week in outdoor activities suffering from anxiety were 0.99 times those with less than one day a week of outdoor activities; however, this finding was statistically insignificant, as the p-value was 0.960 (p>0.05). By keeping the participants ’ fathers’ graduate degrees as the reference category, it was observed that the odds of those whose fathers had studied up to the intermediate level suffering from anxiety were 0.85 times that of those whose fathers had a graduate degree. Likewise, the odds of those participants whose fathers had studied up to middle school suffering from anxiety were 2.08 times higher than those whose fathers had a graduate degree. However, both of these findings were statistically insignificant, as the p-values were 0.323 and 0.121, respectively, which were greater than 0.05. Similarly, by keeping those whose fathers had a business as the reference category, it was observed that the odds of those whose fathers were in other jobs suffering from anxiety were 0.56 times those of those whose fathers had a business. However, this finding was statistically significant, with a p-value of 0.013 (<0.05). Similarly, the odds of those whose fathers were in professional jobs of suffering from anxiety were 0.71 times those than those whose fathers had a business; this finding was also found to be statistically significant, as the p-value was found to be 0.024 (<0.05); the odds of those whose fathers were in government/private services of suffering from anxiety were 0.67 times those than those whose fathers had a business. Additionally, this finding was statistically significant, with a p-value of 0.007 (<0.05).

**Table 3:** Interplay of various determinants on causation of anxiety Data presented as frequency (%); odds ratios (OR) with 95% confidence intervals derived from univariable and multivariable logistic regression analysis; p<0.05 is considered significant

		Anxiety present	No anxiety	OR (univariable)	OR (multivariable)
Gender	Female	323 (54.0)	275 (46.0)	-	-
	Male	426 (52.3)	388 (47.7)	1.07 (0.87-1.32, p = 0.532)	1.07 (0.86-1.33, p = 0.528)
Outdoor activity	<1 d/w	115 (53.0)	102 (47.0)	-	-
	1-4 d/w	301 (52.9)	268 (47.1)	1.00 (0.73-1.37, p = 0.981)	0.97 (0.71-1.33, p = 0.842)
	≥5 days a week	333 (53.2)	293 (46.8)	0.99 (0.73-1.35, p = 0.960)	0.97 (0.71-1.33, p = 0.857)
Father education	Graduate	647 (52.8)	578 (47.2)	-	-
	Upto intermediate	95 (56.9)	72 (43.1)	0.85 (0.61-1.17, p = 0.323)	0.81 (0.58-1.13, p = 0.212)
	Upto middle	7 (35.0)	13 (65.0)	2.08 (0.85-5.56, p = 0.121)	1.59 (0.63-4.35, p = 0.336)
Father occupation	Business	130 (45.3)	157 (54.7)	-	-
	Others	62 (59.6)	42 (40.4)	0.56 (0.35-0.88, p = 0.013)	0.57 (0.35-0.89, p = 0.015)
	Professional	239 (53.8)	205 (46.2)	0.71 (0.53-0.96, p = 0.024)	0.71 (0.52-0.96, p = 0.027)
	Private/government service	318 (55.1)	259 (44.9)	0.67 (0.51-0.90, p = 0.007)	0.68 (0.51-0.91, p = 0.010)

## Discussion

Maintaining good mental health is essential for adolescents, particularly in their ability to manage stress and face daily challenges with resilience. In India, the burden of anxiety among adolescents is compounded by factors such as deprivation, stigma surrounding mental health issues, and a lack of trained mental health professionals. These barriers often delay diagnosis and treatment, making anxiety an even more alarming concern for young people in the country [10].

The current study aimed to assess the prevalence of anxiety among school-going adolescents and found that 53% of the participants experienced some level of anxiety. This is consistent with other Indian studies, which reported a wide range of anxiety prevalence, from 14% to 57% [11-13]. The broad variation across studies may be due to the use of different screening tools, such as the DASS-42, which was used in our study, as well as the timing of data collection. As our data was collected just before the students were to face mid-year and final exams, likely, academic pressures contributed significantly to the elevated levels of anxiety. Exams are a known source of stress and anxiety among adolescents, particularly in a highly competitive academic environment like India. Additionally, the unfamiliarity of the investigator might have contributed to increased anxiety, as participants were required to share personal information with a stranger, further heightening their stress [14].

When comparing anxiety levels by gender, this study found no statistically significant difference, with boys (57%) having slightly higher anxiety rates than girls (43%). This contrasts with many studies that report higher anxiety levels in girls, often attributed to differences in emotional processing and social expectations [15,16]. However, our findings align with studies like those by Bhasin et al., which found no gender predisposition for anxiety [17]. The lack of a significant difference in our study could be due to several factors, including the timing of data collection before exams, which may have resulted in similar levels of academic stress across genders. Additionally, the focus on self-reported data may have led to underreporting or overreporting of anxiety symptoms by either gender, influencing the outcomes.

The age of the participants also did not show a significant association with anxiety levels. However, adolescents, particularly those in the 16-17 age group, are often considered at higher risk for anxiety due to the increasing pressures of academic performance and impending board exams. Studies have shown that anxiety tends to peak during late adolescence, as this is a critical period of transition and decision-making regarding future academic and career paths [18]. Although our results did not show a statistically significant correlation, the trend of higher anxiety in this age group was observed, reinforcing the need for early identification and support during this time.

The findings regarding parental influence were noteworthy. Fathers’ occupation showed a statistically significant association with anxiety, where adolescents whose fathers were in government or private services exhibited higher anxiety levels. This could be attributed to higher expectations placed on these children to excel academically and professionally, given their parents' career backgrounds. High parental expectations, coupled with less time for parental involvement due to demanding work schedules, may exacerbate anxiety among adolescents. In contrast, no significant association was found between mothers’ occupation and anxiety levels. However, mothers who were homemakers or engaged in lower-level occupations were associated with slightly lower levels of anxiety among their children. This suggests that the presence of the mother in the home, or less demanding occupations, may offer more emotional support to adolescents, potentially buffering the impact of anxiety. A comparable pattern was reported by Thomas et al. in a recent cross‐sectional study of 234 rural Tamil Nadu adolescents, where after adjusting for confounders, father’s white-collar occupation was significantly associated with higher scores on social phobia, panic disorder, and generalized anxiety (p<0.05), whereas mother’s employment showed a significant negative association with panic disorder (β=−1.062; p=0.021) [19].

Additionally, the study explored screen time and its impact on anxiety, with the expectation that increased screen time, often associated with reduced physical activity and social interaction, might correlate with higher anxiety levels. However, no significant association was found between screen time and anxiety in this study. This finding is inconsistent with some existing literature that links higher screen time with increased mental health issues, including anxiety, due to factors like decreased self-esteem and academic performance [20,21]. It is possible that adolescents in this study underreported their screen time or that their anxiety was more strongly linked to other stressors, such as academic pressures, than to screen-related activities.

The lack of significant association between outdoor activity and anxiety in this study is also notable. While physical activity is generally seen as beneficial for mental health, including anxiety reduction, our results did not show a clear link. Other studies, such as those by Ussher et al., have found that physical activity positively influences mental well-being by increasing brain activity and improving sleep quality [22,23]. The absence of a significant correlation in our study might be due to participants underreporting their outdoor activities or due to academic stressors outweighing the potential mental health benefits of physical activity.

Finally, the analysis of anxiety symptoms in this study revealed important markers that can help in early identification. Symptoms such as difficulty performing simple tasks, excessive worrying, and feelings of fear became more pronounced as anxiety levels increased. These symptoms are characteristic of GAD and selective mutism, as classified by ICD-10 [24]. The persistence of these symptoms from adolescence into adulthood highlights the importance of early intervention to prevent long-term mental health problems. Studies have shown that anxiety disorders in adolescence often co-exist with other mental health issues and can lead to a vicious cycle of mental health deterioration if left untreated [25-26].

Key strengths of this study include its large, representative urban sample, achieved through rigorous multistage cluster sampling, and the use of a validated bilingual DASS-42 anxiety subscale. These factors together enhance the reliability and cultural relevance of our findings. The link between paternal occupation and adolescent anxiety underscores the importance of school-based screening programs that actively involve both parents and teachers.

Limitations of the study

A notable limitation of our study was its cross-sectional design, which restricted our ability to determine causality. Another limitation of this study is that it was conducted exclusively in an urban setting, limiting its generalizability to adolescents in rural or semi-urban areas. Additionally, as anxiety was assessed through self-reporting, responses might have been influenced by recall bias or social desirability.

## Conclusions

The study found that 53% of the adolescents experienced some level of anxiety, with the father's occupation emerging as a significant determinant. Adolescents whose fathers worked in government or private services exhibited higher levels of anxiety, possibly due to increased expectations and academic pressure. A coordinated approach involving both parents and teachers is required while addressing anxiety in adolescents. Regular collaboration through sessions during parent-teacher meetings could help identify and support students who face anxiety. Teachers should be trained to recognize early signs of anxiety, especially in students vulnerable to external stressors. Schools should be viewed not only as academic institutions but also as places that promote emotional well-being, encourage peer support, and create safe environments where students can openly express their concerns. Future work should adopt longitudinal designs that extend to rural and semi-urban settings and should evaluate interventions aimed at reducing parental occupational stress and enhancing adolescent coping skills.

## Appendices

**Table 4:** Section A questionnaire: general information (including sociodemographic details)

Section A general information (including sociodemographic details)		
Question no.	Question	Response options
1	Unique ID number/ अनोखा आईडी नंबर	__________
2	Age/ उम्र	__________
3	Gender/ लिंग	Boy लड़का / Girl लड़की / Others अन्य
4	Religion/ धर्म	Hindu हिंदू / Muslim मुसलमान / Sikh सिख / Christianity क्रिश्चियानिटी / Others अन्य __________
5	How many hours per day do you spend on computer and mobile phones, including internet use, watching TV programs and playing video games? आप प्रति दिन कितने घंटे कंप्यूटर और मोबाइल फोन पर बिताते हैं, जिसमें इंटरनेट का उपयोग, टीवी कार्यक्रम देखना और वीडियो गेम खेलना शामिल है?	≤2 hours / >2 hours
6	How often do you play outdoor sports per week (minimum 30 minutes per day)? आप प्रति सप्ताह कितनी बार आउटडोर खेल (न्यूनतम 30 मिनट प्रति दिन) खेलते हैं?	Daily रोज / 5-6 days per week प्रति सप्ताह 5-6 दिन / 3-4 days per week प्रति सप्ताह 3-4 दिन / 1-2 days per week सप्ताह में 1-2 दिन / <1 day per week <1 दिन प्रति सप्ताह
7	Do you smoke cigarette or use tobacco? क्या आप सिगरेट पीते हैं या तंबाकू का सेवन करते हैं?	Yes हाँ (______ cigarettes/pouches per day) / No नहीं
8	Do you stay in nuclear or joint family? क्या आप एकल परिवार या संयुक्त परिवार में रहते हैं?	Nuclear एकल परिवार / Joint संयुक्त परिवार
9	How many members are you having in your family? आपके परिवार में कितने सदस्य हैं?	__________
10(a)	Father’s education/ पिता की शिक्षा	Graduate or postgraduate स्नातक या स्नातकोत्तर / Intermediate or diploma इंटरमीडिएट या डिप्लोमा / High school हाई स्कूल / Middle school मध्य विद्यालय / Primary school प्राथमिक स्कूल / Illiterate निरक्षर
10(b)	Mother’s education/ माँ की शिक्षा	Graduate or postgraduate स्नातक या स्नातकोत्तर / Intermediate or diploma इंटरमीडिएट या डिप्लोमा / High school हाई स्कूल / Middle school मध्य विद्यालय / Primary school प्राथमिक स्कूल / Illiterate निरक्षर
11(a)	Father’s occupation/ पिता का व्यवसाय	Professional (doctor/engineer/teacher/lawyer) पेशेवर (डॉक्टर / इंजीनियर / शिक्षक / वकील) / Pvt/Govt service निजी / सरकारी सेवा / Business व्यापार / Others अन्य ______ / Unemployed बेरोज़गार
11(b)	Mother’s occupation/ मां का व्यवसाय	Professional (doctor/engineer/teacher/lawyer) पेशेवर (डॉक्टर / इंजीनियर / शिक्षक / वकील) / Pvt/Govt service निजी / सरकारी सेवा / Business व्यापार / Others अन्य ______ / Unemployed बेरोज़गार
12(a)	Father’s monthly income/ पिता की मासिक आय	__________
12(b)	Mother’s monthly income/ माँ की मासिक आय	__________
13	Have you ever failed in any class because of which you had to suffer year back? If Yes, in how many class? क्या आप किसी कक्षा में अनुत्तीर्ण हुए है, जिसके कारण आप का साल बर्बाद हुआ है? अगर हाँ तोह कौन से कक्षा में हुए हैं?	Yes हाँ (specify class) / No नहीं

**Table 5:** Section B questionnaire: DASS-42 DASS-42: Depression, Anxiety, and Stress Scale

Section B DASS-42 questionnaire (English-Hindi bilingual)						
Instruction: Please read each statement and circle a number (0, 1, 2, or 3) that best describes how much the statement applied to you over the past week. Rating scale: 0 = did not apply to me at all / बिल्कुल नहीं, 1 = applied to me to some degree or some of the time / कुछ हद तक, 2 = applied to a considerable degree or a good part of time / काफी हद तक, 3 = applied to me very much or most of the time / पूरी तरह से						
S. no	Question (English)	प्रश्न (हिंदी)	0	1	2	3
1	I found myself getting upset by quite trivial things	मैं तुच्छ बातों से ही परेशान हो जाता था।				
2	I was aware of dryness of my mouth	मुझे पता है कि मेरा मुँह सूख जाता था।				
3	I couldn’t seem to experience any positive feeling at all	मुझे सकारात्मक भाव अनुभव नहीं हो रहा था।				
4	I experienced breathing difficulty (e.g., excessively rapid breathing, breathlessness)	मैंने साँस लेने में तकलीफ महसूस की (जैसे कि सामान्य से तेज़ साँस लेना)।				
5	I just couldn’t seem to get going	मैं कुछ करने का मन नहीं बना पाया।				
6	I tended to over-react to situations	मैं स्थितियों पर अत्यधिक प्रतिक्रिया देता।				
7	I had a feeling of shakiness (e.g., legs going to give way)	मुझे लड़खड़ाने का अनुभव हुआ (जैसे, टाँगें साथ छोड़ रही हों)।				
8	I found it difficult to relax	मैंने खुद को रिलैक्स करना कठिन पाया।				
9	I found myself in situations that made me so anxious I was most relieved when they ended	मैं ऐसी परिस्थितियों में होता जो मुझे बहुत चिंतित कर देती थीं और उनके समाप्त होने पर ही राहत मिलती थी।				
10	I felt that I had nothing to look forward to	मैंने अपने जीवन को 'खाली'/निरर्थक महसूस किया।				
11	I found myself getting upset rather easily	मैंने देखा कि आसानी से बहुत परेशान हो जाता हूँ।				
12	I felt that I was using a lot of nervous energy	मुझे ऐसा महसूस हुआ कि मैं बहुत अधिक घबराहट की ऊर्जा खर्च कर रहा हूँ।				
13	I felt sad and depressed	मैंने अपने आपको उदास और दुखी महसूस किया।				
14	I found myself getting impatient when I was delayed in any way (e.g., elevators, traffic)	जब मुझे किसी वजह से देर होती थी, मैं बहुत बेचैन हो जाता था।				
15	I had a feeling of faintness	मैंने अपने आपको बेहोशी महसूस किया।				
16	I felt that I had lost interest in just about everything	मैंने महसूस किया कि मेरी किसी चीज़ में दिलचस्पी नहीं बची है।				
17	I felt I wasn’t worth much as a person	मैंने महसूस किया कि मैं ज़्यादा मूल्यवान व्यक्ति नहीं हूँ।				
18	I felt that I was rather touchy	मैंने महसूस किया कि मुझे गुस्सा जल्दी आ जाता है।				
19	I perspired noticeably (e.g., hands sweaty) in the absence of high temperatures or exertion	बिना किसी तापमान या मेहनत के मैं पसीने से तर हो जाता था।				
20	I felt scared without any good reason	मैंने बिना किसी कारण के डर महसूस किया।				
21	I felt that life wasn’t worthwhile	मैंने महसूस किया कि जीवन बेकार है।				
22	I found it hard to wind down	मैंने आराम पाना कठिन पाया।				
23	I had difficulty in swallowing	मुझे निगलने में कठिनाई हुई।				
24	I couldn’t seem to get any enjoyment out of the things I did	मुझे जो कर रहा था उसमें कोई आनंद नहीं मिला।				
25	I was aware of the action of my heart in the absence of physical exertion (e.g., heart racing, skipping a beat)	शारीरिक परिश्रम के बिना भी मैंने अपनी हृदय गति को महसूस किया (जैसे तेज़ धड़कन या रुक-रुक कर धड़कना)।				
26	I felt down-hearted and blue	मैंने अपने आपको दुखी और नीला महसूस किया।				
27	I found that I was very irritable	मैंने महसूस किया कि मैं बहुत चिड़चिड़ा हो गया हूँ।				
28	I felt I was close to panic	मैंने आतंकित महसूस किया।				
29	I found it hard to calm down after something upset me	जब कोई चीज़ मुझे बहुत परेशान कर देती थी, तो मुझे शांत करना मुश्किल होता था।				
30	I feared that I would be “thrown” by some trivial but unfamiliar task	मुझे डर था कि कोई छोटी और अनजानी कार्य मुझे हिला कर रख देगा।				
31	I was unable to become enthusiastic about anything	किसी भी चीज़ को लेकर मुझे उत्साह महसूस नहीं होता था।				
32	I found it difficult to tolerate interruptions to what I was doing	जब मेरे किसी कार्य में रुकावट आती थी, मैं उसे सहन नहीं कर पाता था।				
33	I was in a state of nervous tension	मैं अत्यधिक घबराहट की स्थिति में था।				
34	I felt I was pretty worthless	मैंने महसूस किया कि मैं बहुत बेकार हूँ।				
35	I was intolerant of anything that kept me from getting on with what I was doing	किसी चीज़ की वजह से कार्य में रुकावट आती थी तो मैं बहुत असहिष्णु हो जाता था।				
36	I felt terrified	मैं बहुत डर गया था।				
37	I could see nothing in the future to be hopeful about	मुझे भविष्य में कुछ भी आशाजनक नहीं दिखता था।				
38	I felt that life was meaningless	मुझे लगा कि जीवन निरर्थक हो गया है।				
39	I found myself getting agitated	मैंने अपने आप को अत्यधिक व्याकुल महसूस किया।				
40	I was worried about situations in which I might panic and make a fool of myself	मैं उन स्थितियों को लेकर चिंतित था, जिसमें मुझे पैनिक हो सकता था और शर्मिंदगी उठानी पड़ती।				
41	I experienced trembling (e.g., in the hands)	मैंने कंपकंपी महसूस की (जैसे – हाथों में)।				
42	I found it difficult to work up the initiative to do things	किसी भी कार्य को शुरू करने की मुझे इच्छा नहीं होती थी।				
